# Ancient events and climate adaptive capacity shaped distinct chloroplast genetic structure in the oak lineages

**DOI:** 10.1186/s12862-019-1523-z

**Published:** 2019-11-04

**Authors:** Mengxiao Yan, Ruibin Liu, Ying Li, Andrew L. Hipp, Min Deng, Yanshi Xiong

**Affiliations:** 1 Shanghai Chenshan Plant Science Research Center, Chinese Academy of Sciences, Shanghai Chenshan Botanical Garden, Shanghai, 201602 China; 2 Southeast Asia Biodiversity Research Institute, Chinese Academy of Sciences, Yezin, Nay Pyi Taw, 05282 Myanmar; 3 College of Life Sciences, Shanghai Normal University, Shanghai, 200234 China; 4 The Ecological Technique and Engineering College, Shanghai Institute of Technology, Shanghai, 201418 China; 5 The Morton Arboretum, 4100 Illinois Route 53, Lisle, IL 60532 USA; 6 The Field Museum, 1400 S Lake Shore Drive, Chicago, IL 60605 USA

**Keywords:** *Quercus*, Spatial genetic structure, Climate, Geography, Local adaptation, Chloroplast genome

## Abstract

**Background:**

Understanding the origin of genetic variation is the key to predict how species will respond to future climate change. The genus *Quercus* is a species-rich and ecologically diverse woody genus that dominates a wide range of forests and woodland communities of the Northern Hemisphere. *Quercus* thus offers a unique opportunity to investigate how adaptation to environmental changes has shaped the spatial genetic structure of closely related lineages. Furthermore, *Quercus* provides a deep insight into how tree species will respond to future climate change. This study investigated whether closely related *Quercus* lineages have similar spatial genetic structures and moreover, what roles have their geographic distribution, ecological tolerance, and historical environmental changes played in the similar or distinct genetic structures.

**Results:**

Despite their close relationships, the three main oak lineages (*Quercus* sections *Cyclobalanopsis*, *Ilex*, and *Quercus*) have different spatial genetic patterns and occupy different climatic niches. The lowest level and most homogeneous pattern of genetic diversity was found in section *Cyclobalanopsis*, which is restricted to warm and humid climates. The highest genetic diversity and strongest geographic genetic structure were found in section *Ilex*, which is due to their long-term isolation and strong local adaptation*.* The widespread section *Quercus* is distributed across the most heterogeneous range of environments; however, it exhibited moderate haplotype diversity. This is likely due to regional extinction during Quaternary climatic fluctuation in Europe and North America.

**Conclusions:**

Genetic variations of sections *Ilex* and *Quercus* were significantly predicted by geographic and climate variations, while those of section *Cyclobalanopsis* were poorly predictable by geographic or climatic diversity*.* Apart from the different historical environmental changes experienced by different sections, variation of their ecological or climatic tolerances and physiological traits induced varying responses to similar environment changes, resulting in distinct spatial genetic patterns.

## Background

Anthropogenic climate change has a marked impact on both species and ecosystems throughout the world [1, 2]. Contemporary spatial patterns of population genetic structure are products of (1) modern climate, landscapes, and range shift [3–8], (2) historical factors such as plate tectonics (e.g., orogeny) and climate change (e.g., the glacial-interglacial cycle) [9–11], and (3) species-specific or lineage-specific attributes such as climatically-selected physiological traits, which shape species distribution and their ability to adapt [12–14]. Understanding the mechanisms that generate and structure population genetic variation will help to predict species responses to future climatic conditions and conserve diversity [15, 16]. Furthermore, this will help to unravel the details of how geography and environment influence spatial genetic diversity patterns, which is a central goal of ecological and conservation genetics [17, 18].

Climate change may create particular problems for tree species. While, trees tend to be capable of high rates of local adaptation [19], their long generation times and immobility render them vulnerable to rapid environmental change [1, 20, 21]. Trees, and, oaks (*Quercus*, Fagaceae) in particular are an important group to understand the impacts of environmental change on population genetic diversity for organisms with different traits and distributions [5, 20, 22]. Oaks are among the most widespread woody genera in the Northern Hemisphere, with important ecological functions and economical services for both ecosystem and humans [23–26]. With ca. 450 species, oaks are rich in life history strategies and ecological diversity [27–30]. They dominate a wide range of forest and woodland ecosystems, ranging from temperate deciduous forests and savannas of North America, Europe and Asia, to Mediterranean and desert scrub forests of the Americas and Europe, and to tropical montane forests of South America and Southeast Asia [31]. Moreover, oak seeds are typically recalcitrant (less drought-tolerant and thus limited in their longevity) and their long-range dispersal depend on mammals or birds [32]. As a consequence, the maternal contribution to the population genetic structure, which we can assess directly through plastome sequencing, tends to exhibit conservative, regionalized patterns of diversity [33, 34] that tend to be linked to climate [35]. Hence, oaks offer an ideal system for investigating how natural selection, environmental changes and spatial patterns of existing plants may shape genetic structure of closely related lineages. Due to their wide distribution and dominance, oaks can provide a deep insight into how geological events and climate changes imprint the evolutionary history of forests of the Northern Hemisphere.

Oak plastome haplotypes have been widely sampled, particularly in three major clades: sections *Cyclobalanopsis* and *Ilex*, which have an East and Southeast Asian and Eurasian (sub) tropical distribution, respectively; and section *Quercus*, which is widely distributed in the Northern Hemisphere [27]. Recent phylogeographic studies of the species of section *Cyclobalanopsis* [36–38] indicated that landscape, climate and local adaptation shape regional genetic diversity patterns. Similarly in section *Ilex*, geomorphological and climate changes from the Neogene onward have been demonstrated to have shaped the genetic structure [39–47]. The European white oaks of section *Quercus* have been particularly well studied [33, 48, 49], and the results consistently demonstrated that geographic patterns affect plastome diversity across species boundaries: consequently, it is where populations grow rather than which species they belong to that shapes their plastid haplotype diversity. The impacts of geography, geology and climate on North American members of the section have been explored by less studies [29, 50–53], and with less conclusive findings: while species play a very small role in structuring plastome haplotype diversity, and geography is also not clearly associated in these studies. These studies point to the potential for multiple species to share plastomes, suggesting the potential power in cross-species studies of plastome diversity within oak clades. However, these studies are all restricted to species and clade. This limits the degree to which they can use cross-species, cross-clade comparative analyses to address the relative contributions of the environment and geography to the spatial-temporal pattern of genetic diversity in oaks.

This study addressed two interrelated sets of questions. First, do the closely related oak clades share a similar spatial genetic structure? If not, what induced the distinct genetic patterns of oak clades? Second, what roles have geographic distribution, ecological tolerance and historical environmental changes played in the genetic diversity of each clade? This study sampled plastome haplotypes of representative species from three oak clades (sections *Cyclobalanopsis*, *Ilex* and *Quercus*) that span the geographic range of each. We aim to figure out the underlying mechanisms that influence the maternal genetic pattern of oaks to predict how future climatic changes will affect acorn migration and to provide new insights into the response of trees to future climate change, and to offer suggestions for the sustainable management of forest tree populations.

## Results

### Genetic diversity and neutrality tests

**Fig. 1:**
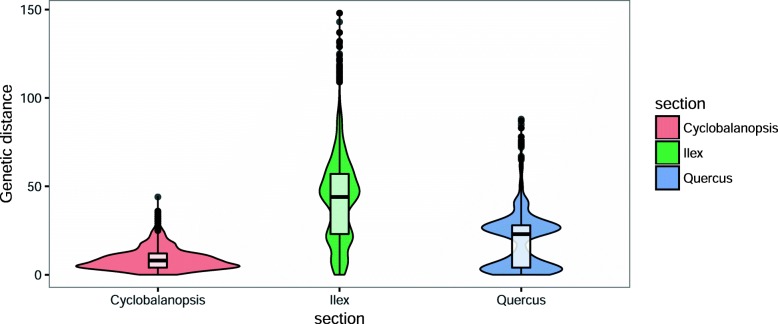
Distribution of pairwise genetic distances among accessions of sections *Cyclobalanopsis*, *Ilex* and *Quercus*

Section *Cyclobalanopsis* exhibited the lowest mean *p*-distance (0–0.0020), nucleotide diversity (0.917) and Fst (0.3220) among the three sections (Table 1). In addition, the majority of its pairwise genetic distances were narrowly distributed at low values (Fig. 1). Section *Ilex* had the highest genetic diversity as estimated using *p*-distance (0–0.0090), genetic distances and nucleotide diversity (0.985), however, it had a median Fst of 0.4624 (Fig. 1, Table 1). The genetic diversity of section *Quercus* was in-between (Fig. 1). Its *p*-distance (0–0.0046), genetic distances and nucleotide diversity (0.934) were medium (Fig. 1, Table 1), however, section *Quercus* had the highest Fst of 0.7436.

**Table 1:** Genetic diversity parameters of three sections of genus *Quercus*

Sections	N	Ns	*p*-distance	Vs		Pi	Hn	Hd	Indel	Fst
				number	%					
*Cyclobalanopsis*	147	28	0–0.019 (0.0020 ± 0.0031)	145	4.87	0.00252	54	0.917	56	0.3220
*Ilex*	121	28	0–0.018 (0.0090 ± 0.0041)	163	5.27	0.01289	66	0.985	184	0.4624
*Quercus*	89	27	0–0.020 (0.0046 ± 0.0014)	77	2.61	0.00415	30	0.934	90	0.7436

***N*** number of samples, ***Ns*** number of species, ***p-distance*** (min.–max. (mean ± SD)), ***Vs*** variable sites, ***Pi*** nucleotide diversity, ***Hn*** number of haplotypes, ***Hd*** haplotype diversity

Tajima’s *D*, Fu and Li’s *F* and Fay and Wu’s H were significantly negative on section *Cyclobalanopsis* (Table 2), suggesting a recent selective sweep in section *Cyclobalanopsis*. Neutrality tests for section *Ilex* were nonsignificant. Fu and Li’s *D* was moderately significantly (*p* < 0.05) negative and Fay and Wu’s H was negative in section *Quercus*. This is likely the result of population shrinkage during the glacial period.

**Table 2:** Neutrality test of sections *Cyclobalanopsis*, *Ilex* and *Quercus*

Section	Tajima’s *D*	Fu and Li’s *D*	Fu and Li’s *F*	Fay and Wu’s *H*
*Cyclobalanopsis*	−2.35202**	−1.95271	−2.55835*	− 27.2651
*Ilex*	−1.01241	−0.44143	−0.83735	1.7057
*Quercus*	−0.92330	−2.34890*	−2.10962	−12.4408

* *p* < 0.05; ** *p* < 0.01

### Phylogenetic and spatial structures

**Fig. 2:**
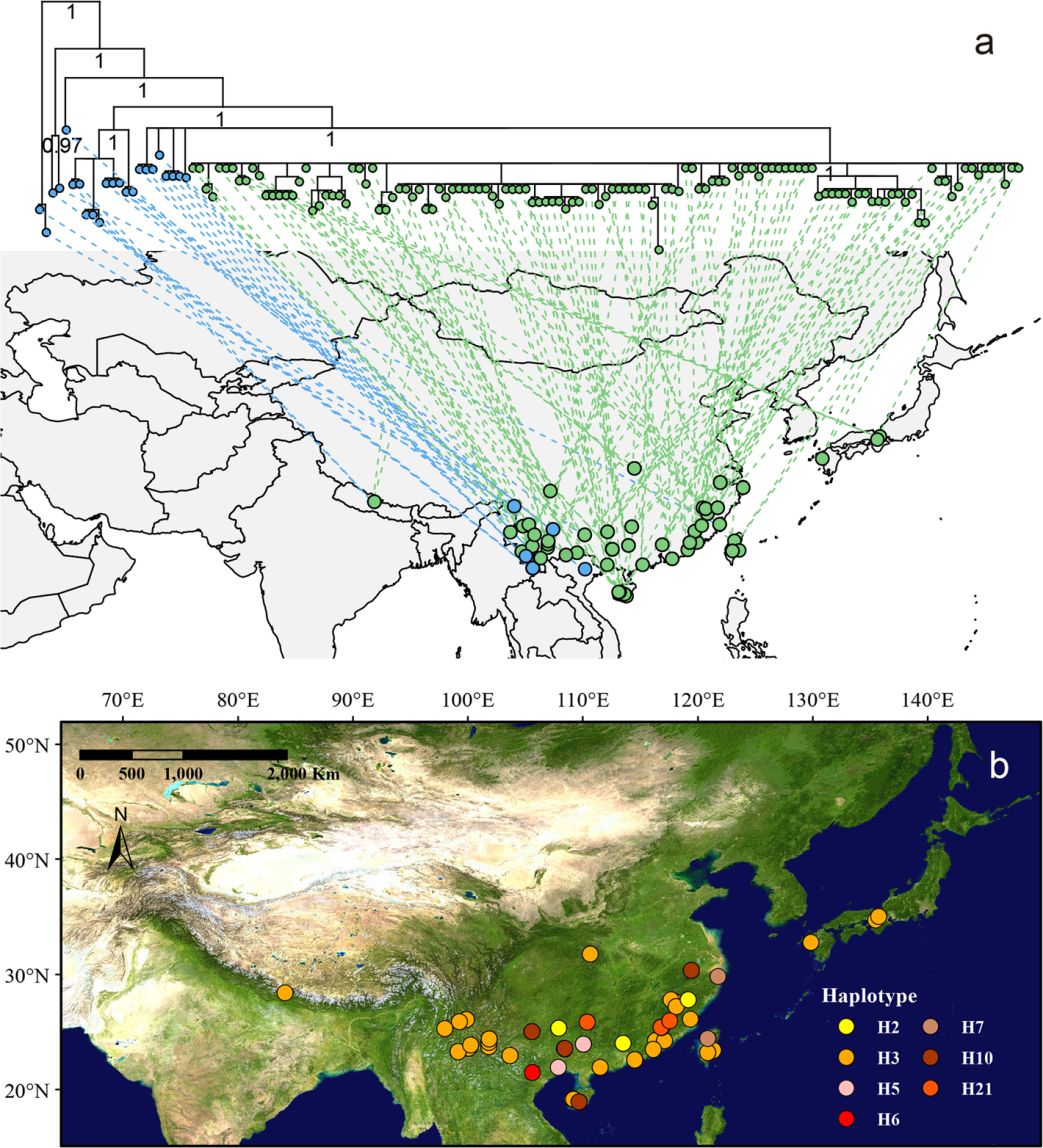
Phylogeny of haplotypes and shared haplotypes of section *Cyclobalanopsis*. **a**. Bayesian inference phylogram and geographic distribution of section *Cyclobalanopsis*; **b**. Geographic distribution of interspecific shared haplotypes of section *Cyclobalanopsis*

The earliest diverging branches of section *Cyclobalanopsis* (blue dots in Fig. 2a) were concentrated in southwest China (SW China), Vietnam and Nepal. These areas are near the western edge of the range of section *Cyclobalanopsis*, but do not show any clear geographic structure. The majority of individuals in this section formed a large lineage encompassing the entire range of the section (green dots in Fig. 2a), which are widespread in subtropical East Asia, including Japan, mainland China and even stretching to Nepal. This main lineage likewise did not exhibit geographic structure. In total, seven haplotypes were observed in this section (H2- H3, H5-H7, H10, and H21; Fig. 2b), which were all shared among species. Haplotypes H5 and H6, were restricted to SW China, while all others were geographically widespread (Fig. 2b). Particularly haplotype H3 essentially spaned the entire geographic distribution of the section, from the East Asian subtropics to Northern Indochina and the southern slope of the Himalayas.

**Fig. 3:**
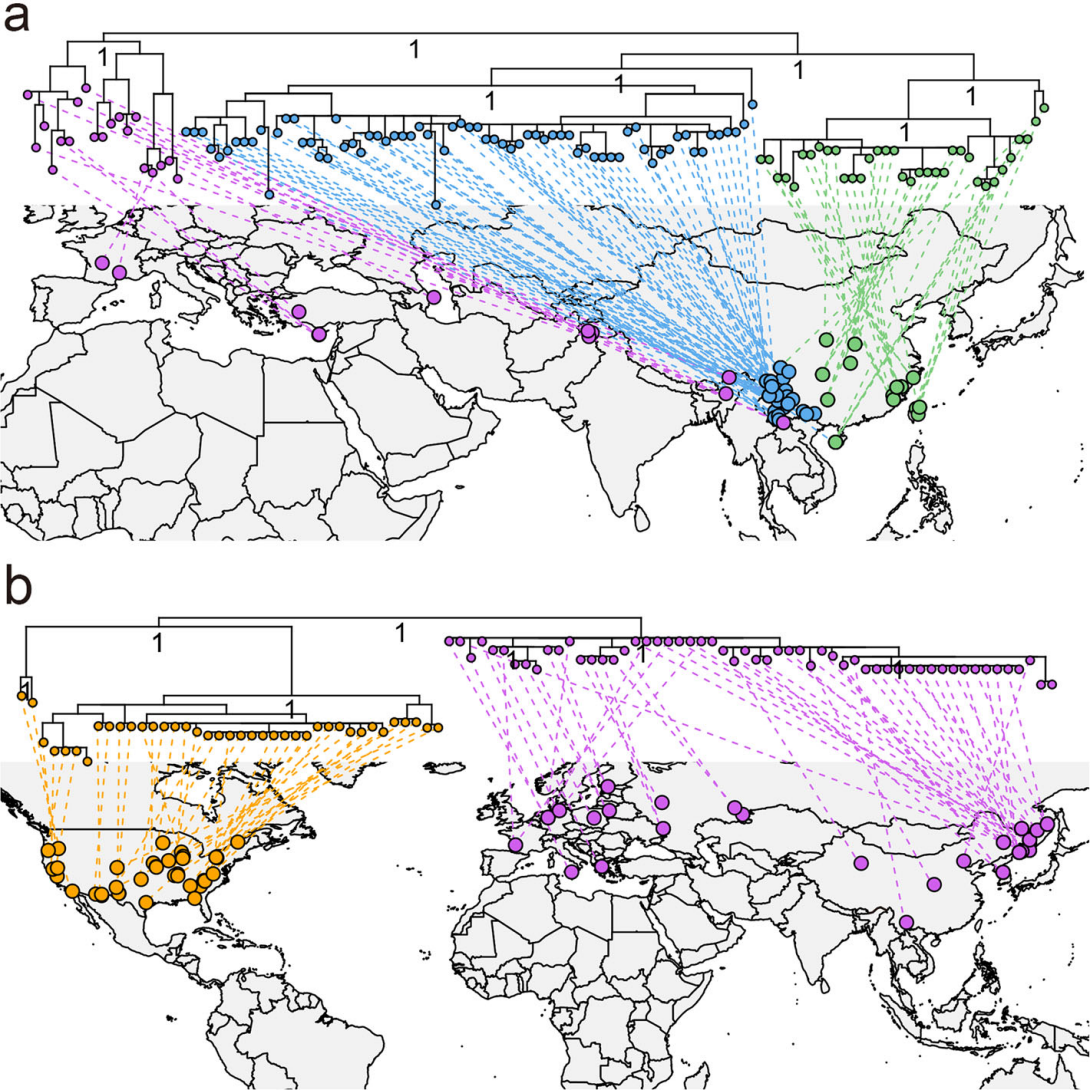
Bayesian inference phylogram and geographic distribution of section *Ilex* (**a**) and section *Quercus* (**b**)

The chloroplast phylogeny of section *Ilex* showed a strong geographic structure with the Himalayas-Mediterranean lineage (purple dots in Fig. 3a) strongly separated from the East Asian lineage. The East Asian lineage further separated into the SW China lineage (blue dots in Fig. 3a) and the Sino-Japan lineage (green dots in Fig. 3a), comprising individuals from the tropics and subtropics of both mainland China and Taiwan Island. The sharing of haplotypes among species (H68, H81, H89, H91, H96, H99 and H114) in this section was also identified and these interspecific haplotypes were locally distributed (Additional file 1: Table S1, Additional file 2a).

Section *Quercus* was composed of two lineages, corresponding to the Eurasian lineage (purple dots in Fig. 3b) and the North American lineage (orange dots in Fig. 3b). Within regions, however, extensive sharing of haplotypes (H42–H43, H47–H50, H56–H58, H62 and H65) were observed among species of the section (Additional file 1: Table S1, Additional file 2b). This has also been reported in previous studies [33, 50, 53].

### Climatic niches

To identify which climatic variables restrict the geographic distribution of different sections, the contribution of climatic variables was ranked (Additional file 1: Table S2). The top climatic factors that restricted species distribution in section *Cyclobalanopsis* were bio12 (annual precipitation), bio3 (isothermality), bio1 (annual mean temperature), and bio7 (temperature annual range), in the order of variable contribution. For section *Ilex*, the top climatic factors with the highest contributions were bio7, bio3, bio1 and bio12. Furthermore, for section *Quercus,* they were bio1, bio12, bio3 and bio19 (precipitation of coldest quarter). Overall, annual mean temperature and annual precipitation were key climatic variables that restricted species distribution of the three oak sections.

**Fig. 4:**
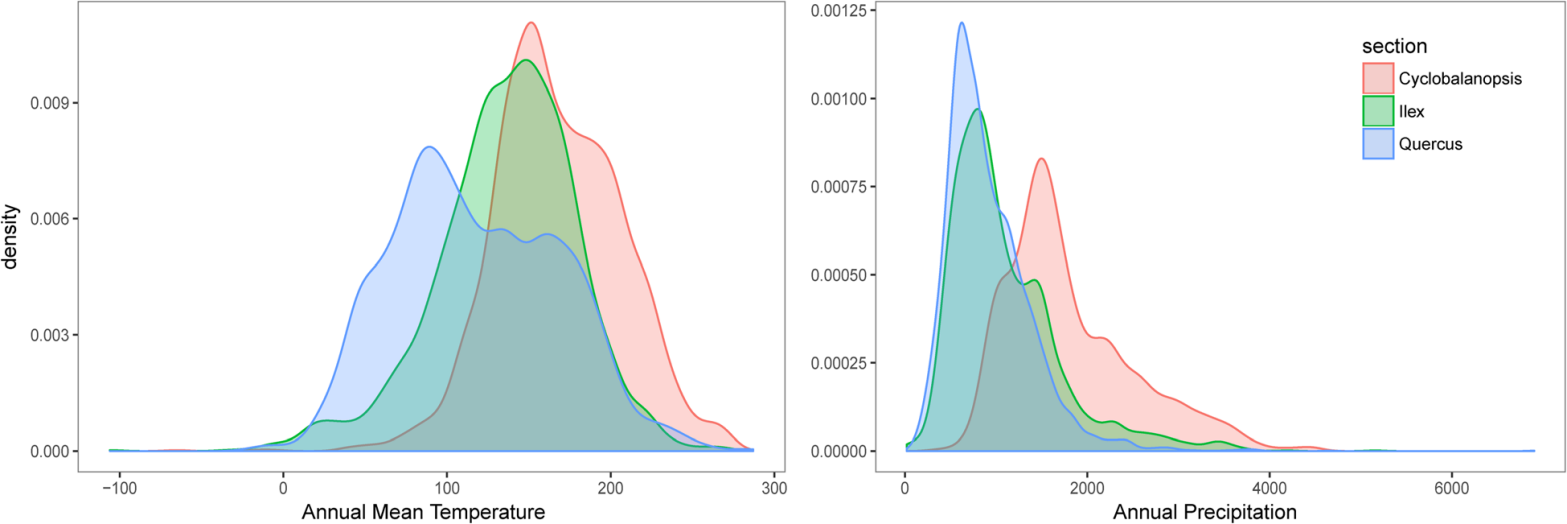
Density curve of restricting climatic factor of sections *Cyclobalanopsis*, *Ilex* and *Quercus*

**Fig. 5:**
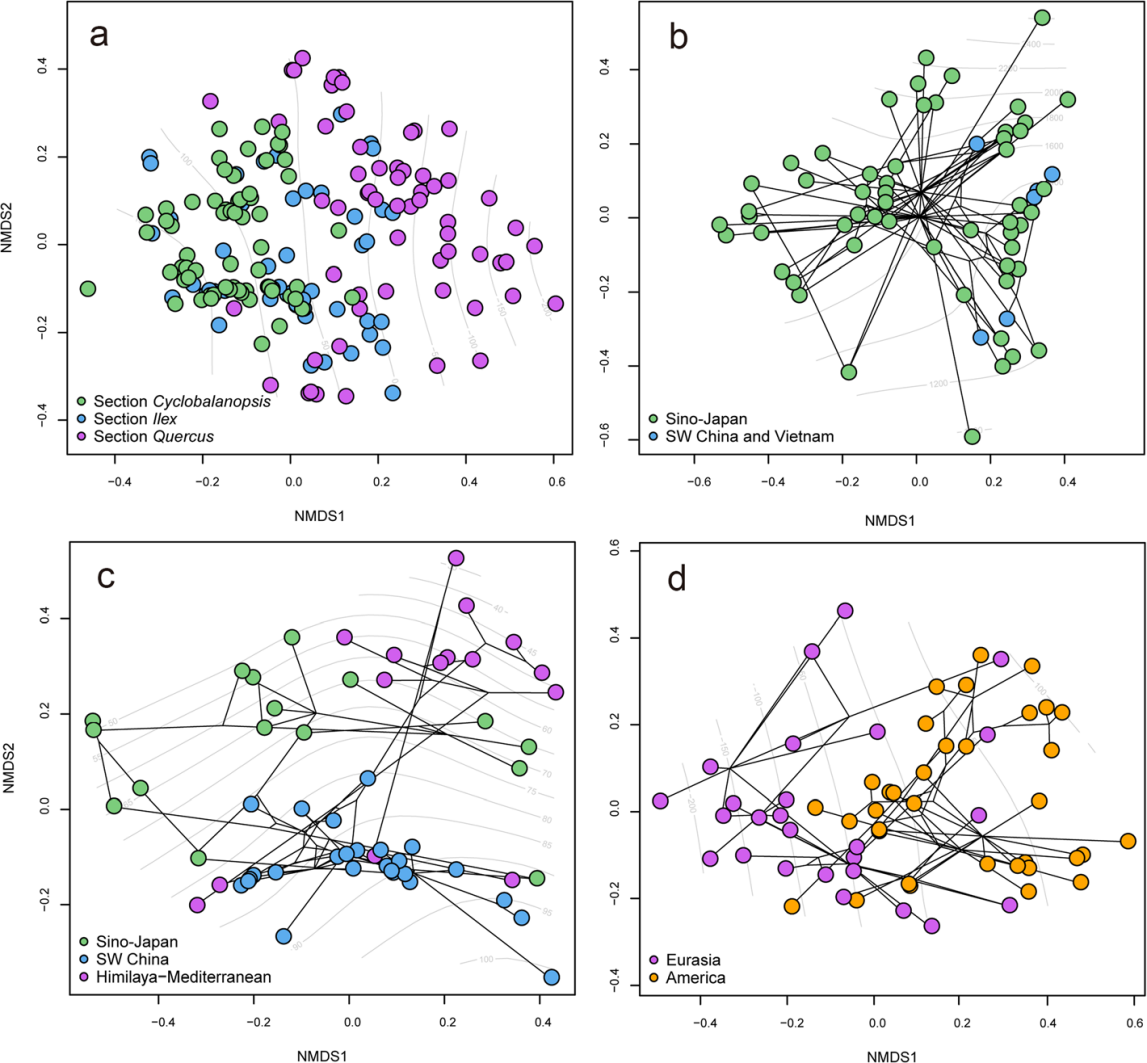
Nonmetric multidimensional scaling (NMDS) analysis based on Gower distances for 19 BIOCLIM variables, averaged over species. Points in all panels represent species; line segments between points represent phylogenetic branches, and node position were estimated using generalized least squares weighted means for the ancestral states. Panels represent (**a**) all three sections, (**b**) section *Cyclobalanopsis*, (**c**) section *Ilex*, and (**d**) section *Quercus*

Section *Cyclobalanopsis* had relatively narrow ranges of annual mean temperature and annual precipitation (Fig. 4), corresponding to low multidimensional climatic niche diversity (Fig. 5a). It had higher mean annual temperature than section *Quercus*, and higher mean annual precipitation than both sections *Quercus* and *Ilex* (Fig. 4). This indicates their adaption to a rather narrowly warm and humid environment compared with the other two sections. In contrast, section *Ilex* was distributed in areas with moderate annual mean temperature and both humid to arid regions (Figs. 4, 5a). The transcontinental section *Quercus* had the widest annual mean temperature range (Fig. 4, 5a), adapted to the winter cold and aridity of Eurasia and northern North America, which corresponding to the highest multidimensional climatic niche diversity (Fig. 5a).

### Effect of geography and climate on the spatial genetic diversity pattern

Nonmetric multidimensional scaling (NMDS) analysis of section *Cyclobalanopsis* showed limited climatic differentiation between the SW China lineage and the Sino-Japan lineage (Fig. 5b). In contrast, three main climatic clusters were identified within section *Ilex*, corresponding to Sino-Japan, SW China and Himalaya-Mediterranean, which was consistent with its phylogenetic and geographic structure (Fig. 5c). Similarly, section *Quercus* segregated into largely Eurasian and North American clusters in the climatic space with few intermediates (Fig. 5c).

Genetic distances within section *Cyclobalanopsis* and its two lineages were poorly predicted by either geographic or climatic diversity (*r*^2^ in all models > 0.05; Table 3). In contrast, genetic distances of section *Ilex* were significantly predicted by geographic distances (*r*^2^ = 0.3603, *p* = 0.001) but not by climate (Table 3). Genetic distances within the Sino-Japan and the Himalayas-Mediterranean lineages were predicted well by geography and climate. However, the SW China lineage showed weak correlation between genetic distances and geography (r^2^ = 0.033, *p* = 0.022) and no correlation with climate (Table 3). Geographic distance (r^2^ = 0.3713, *p* = 0.001) and climatic distances (r^2^ = 0.1458, *p* = 0.001) were both strong predictors of genetic variation within section *Quercus*. Within geographic lineages, where plastomes were shared relatively freely, North American genetic distances were well predicted by geography (r^2^ = 0.1471, *p* = 0.001) and climate (r^2^ = 0.1654, *p* = 0.001), however, the Eurasian lineage showed no significant correlations (Table 3).

**Table 3:** Mantel test of the relationship between genetic distances and geographic and climatic distances in *Quercus* sections *Cyclobalanopsis*, *Ilex*, and *Quercus* (**Him-Med**: the Himalayas-Mediterranean)

Sections	Lineage	Genetic~geo	Genetic~ clim	Genetic~ geo + clim
*Cyclobalanopsis*	all	r^2^ = 0.0005	r^2^ = 0.0007	r^2^ = 0.0007
	Sino-Japan	r^2^ = 0.0005	r^2^ = 0.0008	r^2^ = 0.0009
	SW China	r^2^ = 0.0144	r^2^ = 0.0122	r^2^ = 0.0155
*Ilex*	all	r^2^ = 0.3603 **	r^2^ = 0.0038	r^2^ = 0.3608 **
	Sino-Japan	r^2^ = 0.1563 **	r^2^ = 0.2363 **	r^2^ = 0.2390 **
	SW China	r^2^ = 0.0337 *	r^2^ = 0.0178	r^2^ = 0.0337
	Him-Med	r^2^ = 0.1333 *	r^2^ = 0.1546 *	r^2^ = 0.1661
*Quercus*	all	r^2^ = 0.3713 **	r^2^ = 0.1458 **	r^2^ = 0.3880 **
	Eurasia	r^2^ = 0.0293	r^2^ = 0.0088	r^2^ = 0.0223
	North America	r^2^ = 0.1471 **	r^2^ = 0.1654 **	r^2^ = 0.1799 **

* *p* < 0.05; ** *p* < 0.01

## Discussion

### Evolution of section *Cyclobalanopsis*

Although section *Cyclobalanopsis* originated from comparable ages as sections *Ilex* and *Quercus*, which is dated to Oligocene [29, 54], the individuals of section *Cyclobalanopsis* show very high genetic similarity and low genetic diversity. Haplotypes are shared widely among species (Additional file 1: Table S1), and most interspecifically shared haplotypes are geographically widespread: haplotype H3 shows particularly wide geographic range and interspecies sharing (Fig. 2b). Haplotype sharing among species across large geographic distances could be a result of incomplete lineage sorting [55–57]. In turn, incomplete lineage sorting could result from rapid radiation during early diversification, random sharing or extinction of genotypes, and/or extremely large effective population sizes [58]. In section *Cyclobalanopsis*, interspecific shared haplotypes appear to postdate the early diversification of the lineage, thus making this explanation relatively unlikely. Moreover, the chloroplast DNA is haploid; therefore, its effective size is very small [59]. Furthermore, the haplotypes of section *Cyclobalanopsis* are not randomly shared. Haplotype H3 is shared by 50% of individuals, and interspecific shared haplotypes (H2–H3, H5–H7, H10, and H21) are shared by 69.6% of individuals. Therefore, incomplete lineage sorting is unlikely explain the large-scale sharing of haplotypes.

Alternatively, the high genetic similarity and large-scale haplotype sharing that was observed within section *Cyclobalanopsis* could be a result of selective sweeps on the chloroplast. This is supported by the results of the conducted neutrality tests (Table 2). Significantly negative Tajima’s *D* and Fu and Li’s *F* (based on coalescent) indicate a recent selective sweep, as well as population expansion after a recent bottleneck [60]. However, Fay and Wu’s H is suited to distinguish variation due to recent population expansion or direction sweep [60]. A negative Fay and Wu’s H indicates an excessive high-frequency derived single nucleotide polymorphisms [61], suggesting a recent selective sweep in section *Cyclobalanopsis*. The majority of individuals in this study (viz, the Sino-Japan lineage) are genetically similar and dominated by few haplotypes, which is exactly as expected under purifying selection. The chloroplast genome can undergo trans-specific selective sweeps, as previously reported, e.g., in *Salix* [58], *Begonia* [62] and *Silene* [63]. Weak reproductive isolation in section *Cyclobalanopsis* [64–66], likely facilitates plastid capture [67, 68], which is a first step in spread of a selected plastome.

Both the timing and geography in section *Cyclobalanopsis* could then have intensified the selection for attenuated plastome diversity. Diversification and range expansion of section *Cyclobalanopsis* from SW China-Indochina to East Asia were driven by a combination of climatic and orogenic factors, initiated by the extrusion of Indochina since the Oligocene [69–71] and an intensification of East Asian monsoon [72] since the Miocene [36, 73]. The current distribution of section *Cyclobalanopsis* is relatively uniform in climate. Section *Cyclobalanopsis* is a dominant woody lineage of warm and humid Asian (sub) tropical evergreen broadleaved forests [73] (Fig. 4, 5b). Thus, the history of diversification combined with the present distribution may have selected for narrowed plastid diversity, since many functionally important genes are encoded by the chloroplast [68]. Moreover, a cyto-nuclear linkage disequilibrium has been reported in plants [74, 75]. Therefore, selection for nuclear genes may also have resulted in selective sweeps on the plastid. While the identified phylogeny shows no clear correlation with climate (Fig. 5b, Table 3), it remains to be seen whether any functional plastome variation is associated with climatic gradients in this section.

### Evolution of section *Ilex*

While the genetic homogeneity of section *Cyclobalanopsis* implicates selection as a driving force in its plastid evolution, section *Ilex* exhibits the hallmarks of geographic isolation. Its three lineages correspond to Sino-Japan, SW China and the Himalayas-Mediterranean. Moreover, geography explains a significant proportion of genetic diversity both among (*r*^2^ = 0.3603) and within (*r*^2^ = 0.0337–0.1563) these lineages (Table 3), supporting previous findings in favor of plastid geographic differentiation in this section [40, 43, 47]. Geographic isolation in section *Ilex* is strongly congruent with geological and paleobotanical data. Fossils of section *Ilex* in Oligocene-Miocene sediments from East Asia [76, 77], Himalayas [78, 79], Asian minor [80, 81], Middle East [80, 81] and Mediterranean [82–86] suggest that the ancestral lineage of section *Ilex* was once widespread throughout East Asia and Tethys-Paratethys. This widespread lineage was then segmented gradually. Initially, orogeny of the Himalayas beginning in the Middle Eocene [87–90] could explain the split between the Asian lineage and the Himalayas-Mediterranean lineage [39]. The uplift of the Hengduan Mountains and the associated cooling from the middle Miocene onward, appears to have triggered the divergence of the Sino-Japan lineage and the SW China lineage [40, 47]. These barriers are particularly likely to have affected plastid genetic diversity, since the chloroplast is maternally inherited in oaks [91], and Eurasian dispersal of oak acorns is mainly by gravity, stream, rodents and Corvids [92–94], which is not very efficient for a long distance dispersal for a species with highly fragmented distribution. Moreover, the seeds of oaks are typical recalcitrant, and thus have a very short viability if they become dessicated [95], which further limits their potential dispersal range. As a result, physical barriers encountered by section *Ilex* were particularly effective at differentiation of the main lineages.

Local adaptation may also have shaped the distribution of plastid diversity as seen in this section. Section *Ilex* has high ecological diversity, ranging from semi-savanna maquis in pan-Mediterranean, semi-arid subalpine scrubs and deserts of the Himalayas, and evergreen broadleaved forests of East Asia and Northern Indochina [42]. This work also demonstrates that section *Ilex* has a wide climatic niche (Figs. 4, 5a). Meanwhile, individuals of section *Ilex* form corresponding clusters in climatic ordinations (Fig. 5c), suggesting that geographic differentiation may have worked in concert with the climatic selection to shape the genetic divergences of today. In addition, the genetic diversities of the Sino-Japan and Himalayas-Mediterranean lineages can be significantly predicted by both geography and climate (Table 3). Within the Sino-Japan lineage of section *Ilex*, three main sublineages (South China, East China and Central China) were inferred (Fig. 3a). Similarly, three to four distinct sublineages were identified in the individuals of section *Ilex* from the Mediterranean [42, 43]. The main interruptions of haplotype migration coincide with the mountain ranges of Anatolia, Greece and the Balkans, the Sea of Sardinia and the Libyan Sea [43]. It may be that geological barriers resulted in allopatric diversification, and that local adaptation contributed to genetic differentiation within lineages.

### Evolution of section *Quercus*

Section *Quercus* is widely distributed in European and East Asian forests, and in wetlands of the upper Midwestern USA to droughty mountains of the arid southwest. In North American alone, they range from southern Canada to Central America [26, 96, 97]. This study demonstrates that this section exhibits a particularly wide climatic range (Fig. 4, 5b), as has been previously suggested in a sampling of the American taxa [29]. The genetic diversity of section *Quercus*, however, is lower than that of section *Ilex*. This is perhaps a result of the geographic range of white oak was strongly impacted by Quaternary climatic fluctuations. During Quaternary glaciation periods, the continental ice shield covered much of Europe and North America [98], which led to both widespread extinction and biotic shifts. In Europe, white oaks retreated to southern refugia, e.g., Iberian Peninsula, southern Italian Peninsula and the southern Balkan Peninsula [99]. Extensive continental ice dominated North America for the 2.4 Myr of the Pleistocene [100], and the eastern North American oak diversity was limited to the unglaciated southeastern portion of the continent [101–103]. Cold temperatures drove the local extinction of northern populations of these white oak species, presumably reducing genetic diversity. In addition, during inter-glacial and post-glacial recolonization, their genetic diversity decreased due to continuous bottleneck effects [91, 100]. In contrast, the European species of section *Ilex* are mainly distributed around the pan-Mediterranean, where they were less impacted by Pleistocene climatic fluctuations and could thus maintain their population sizes and higher genetic diversity.

The plastomes of the white oak derive from ancient and rapid diversification into four major plastome lineages in sections *Protobalanus* (not sampled here) and *Quercus* in only the plastome data, while nuclear data show clear monophyly of the sections [50, 104]. Moreover, the plastome resolves into reciprocally monophyletic lineages, which corresponding to the Eurasian and American white oaks distribution in section *Quercus*. This result seems most plausibly raised from long-term geographic isolation of the two groups of white oaks due to submersion of the North Atlantic land bridge and Bering land bridge since the late Neogene [105]. Climate cooling [106] and Central Asian aridity [107–109] since the middle Miocene are likely causes the vicariance between the European and East Asian white oak groups.

However, the genetic variation of the Eurasian lineage does not correlate with geography or climate (Table 3). The European white oaks typically exhibite strong geographic structure [33, 49] due to founder effects during post-glacial recolonization [33, 48, 49]. In contrast, the genetic variation of the North American lineage shows a significant correlation to geography and climate (Table 3), which is in large part, due to the geographic and reproductive isolation of the Californian species from the eastern North American species [50]. However, white oaks in Eastern North American do not demonstrate chloroplast genetic structure, presumably due to their capacity for long-distance gene flow, their large population sizes and their relative continuous Pleistocene refugia [50].

### Factors resulting in different spatial genetic structure of sections *Cyclobalanopsis*, *Ilex* and *Quercus*

The disparate spatial genetic patterns of these oak clades appear to result from variance in their ecological / climatic tolerances, migration histories, the strength of climatic selection and physiological traits. Climatic tolerance is likely the easiest of these to characterize. Section *Cyclobalanopsis* is restricted to warm and humid subtropical Asia and has an almost continuous distribution within its range. While section *Ilex* has a wider climatic range, it is still unsuited to the dry and cold climate of central Asia, which shapes its disjunct distribution in East Asian-Himalayas and Mediterranean. White oaks, in contrast, have adapted to a wide spectrum of environments and have the widest geographic distribution, comprising the most diverse and productive (in terms of number of species and total biomass) woody plant genus of North America [25]. From the Cretaceous to the Late Neogene, both the Bering Land Bridge and the North Atlantic Land Bridge served as filters that allowed specific plant taxa to migrate between Eurasia and the Americas [110, 111]. White oaks were well adapted to cool climates and thus distributed at high latitudes. Consequently, they were capable to cross the land bridge(s) during the latest Miocene, as inferred by pollen found on Iceland and Greenland [111]. Thus, the climatic niche breadth of white oaks contributed to their wide distribution in throughout the Northern Hemisphere, in contrast to other sections.

In addition, adaptive traits can induce varying responses to similar environment changes. Several species of section *Ilex* from East Asia share similar geographic distributions with section *Cyclobalanopsis.* Both sections originated during the late Eocene and experienced similar topological and climate changes in the Himalayas-East Asia [54, 73]. However, section *Ilex* exhibits clear geographic differentiation, while section *Cyclobalanopsis* does not. As discussed above, this may be partially explained by selective sweeps, which affect the chloroplast genome of section *Cyclobalanopsis* and results rapid migration of selected haplotypes. However, varying adaptive traits, e.g. reproductive capacity and seed dispersal distance may account for their different spatial genetic structure. The geographic distribution of section *Cyclobalanopsis* is mostly concentrated in low to middle elevation montane areas with rugged topography [112]. Therefore, gene flow is not strongly structured by topography. In contrast, most species of section *Ilex* inhabit highly fragmented landscapes, e.g. sky-island such as subalpine areas or river gorges in mountains [112], which disfavors long distance seed dispersal. Furthermore, sections *Cyclobalanopsis* and *Ilex* have different life-forms and tradeoffs between vegetative and reproductive growth. For example, most species of section *Ilex* are smaller trees and shrubs, and produce fewer seeds than species of section *Cyclobalanopsis* [112], and have prominent mast years. Therefore, maternal gene flow is more limited in section *Ilex* than in section *Cyclobalanopsis*, and more influenced by both local adaptation and genetic drift, thus increasing their genetic spatial structure.

### Comparison of plastome and nuclear genetic diversity patterns

The plastome genetic diversity pattern of *Quercus* does not always match the pattern of nuclear genetic diversity. Section *Cyclobalanopsis* has high species richness [112, 113], which is influenced by environmental variables, mostly by water availability [113]. However, the plastome genetic diversity of section *Cyclobalanopsis* is very low and could not be predicted by climatic factors. While, the spatial phylogenetic structures of sections *Ilex* and *Quercus* were confirmed by nuclear DNA. Based on RADseq nuclear markers, the species of sections *Ilex* from Mediterranean-Himalaya formed a monophyletic clade, though species from Sino-Himalaya and Sino-Japan mixed together [54]. Eurasian white oaks and North American white oaks formed two monophyletic clades, which were supported by RADseq nuclear markers [29, 114]. Furthermore, the nuclear genetic variation of white oaks could partly predict the plastome genetic variation (*r*^2^ = 0.311, *p* < 0.002) [50].

Although nuclear genetic variation may show some similarity to plastome markers, they had very different evolution histories. The chloroplast is maternally inherited in oaks, and can only be transferred through seeds [91], while nuclear DNA is biparentally inherited. Therefore, differences between plastome and nuclear genetic structure reflects the efficiency of seed-induced gene flow and pollen-induced gene flow, respectively, which has also been reported for other oak species as well [39, 115–117]. Moreover, the whole chloroplast genome could be transferred among different species during hybridization processes, since hybridization frequently occurred among oaks of the same section [66, 114, 118]. However, only half of the nuclear homologues can be passed to the offspring. Furthermore, few nuclear genes can penetrate among specie due to the natural selection. As a result, nuclear DNA is less impacted by hybridization. All these factors can lead to different genetic structure between plastome DNA and nuclear DNA in oaks.

## Conclusions

This study clarifies the individual diversification history of the sections *Cyclobalanopsis*, *Ilex*, and *Quercus*, as manifest by their chloroplast diversity. The results highlight the importance of geological events and ecological adaptive capacity for the spatial genetic pattern of oak clades and provides detailed insights into the formation mechanism of their contemporary diversity. Further insights into the divergence history of this groups will originate from a combination of whole-chloroplast sequencing and nuclear genetic data of deeper population sampling. Finally, association mapping can be used to investigate the relationship between genetic polymorphisms and environment, which will help to identify the relative effects of the climatic, edaphic variation, and migration history on genetic variation in multiple clades.

## Methods

### Sampling and molecular biology experiments

This study included 147 individuals from 29 species of *Quercus* section *Cyclobalanopsis* from China, Japan, Vietnam and Nepal as well as 121 individuals of 28 species of section *Ilex* from China, Himalayas, Azerbaijan, and Mediterranean. One individual of *Lithocarpus henryi* was used as outgroup to root the tree of genus *Quercus*. These data were obtained from our previous study [119], including four chloroplast DNA regions: *atp*I-*at*pH, *mat*K, *psb*A-*trn*H and *ycf*1. Eighty-nine individuals from 27 species of section *Quercus* from Eurasia and North America were included in this study. Among these, seven individuals of the six species were obtained from our previous study [119]. The remaining samples (82 individuals and 21 species) were obtained from a previous study [50]. The four *cp*DNA regions were aligned using Muscle [120] implemented in MEGA 7.0.21 [121] (available at http://www.megasoftware.net/) and the extra end of long sequences were trimmed. An inversion of 60 bp found in the *psb*A-*trn*H region was replaced with its reverse complement (to maximize homology in the phylogenetic analyses). Relevant data on samples and sequences used in this study are included in the additional file (Additional file 1: Table S1).

### Genetic diversity and phylogenetic analysis

Haplotypes were extracted by using DnaSP 5.10 [122]. Genetic diversity pattern were profiled using variable sites, average pairwise differences per base pair between sequences (nucleotide diversity) [123], haplotype diversity (Hd), Fst in DnaSP 5.10 [122] and *p*-distances in MEGA 7.0.21 [121] (available at http://www.megasoftware.net). Genetic distances were calculated in GenAlEx 6.5 [124]. Neutrality tests (Tajima’s *D*, Fu and Li’s *D*, Fu and Li’s *F* and Fay and Wu’s H) were conducted using DnaSP 5.10 [122].

Bayesian trees were constructed using MrBayes 3.2.6 [125]. The nucleotide substitution model was selected by Modeltest 3.7 [126] based on the Akaike information criterion (AIC). Two parallel Markov chain Monte Carlo (MCMC) runs were performed for 20 million generations. The trees were sampled every 1000 generations and inspected via Tracer 1.6 (available at http://tree.bio.ed.ac.uk/software/tracer) to ensure effective sample size (ESS) exceeding 200. The first 15% of trees were discarded as burn-in. Phylogenetic trees were plotted on the world map using R package phytools [127].

### Geographic and climatic data

Georeferenced occurrence records were collected for each section from public specimen databases and the publications listed below and were augmented by our own collections. Data were initially cleaned by removing all records that were outside of published range records. Coordinates of these specimens were then used to extract 19 BIOCLIM variables from WorldClim 1.4: Current conditions (~ 1960–1990) [128] using the raster [129] and dismo packages [129] in R. Geographic records of sections *Cyclobalanopsis*, *Ilex* and *Quercus* were obtained from our field collection database, Chinese Virtual Herbarium (http://www.cvh.ac.cn), Global Biodiversity Information Facility (https://www.gbif.org) and National Herbarium of the Netherlands (http://herbarium.naturalis.nl/nhn/explore). Additional geographic occurrence data were added from previous studies for section *Cyclobalanopsis* [36–38, 130], section *Ilex* [39, 43, 47, 131], and section *Quercu*s [29, 50, 130, 132–134]. The sampling records of sections *Ilex* and *Quercus* were uneven, which would have affect climate analysis. Therefore, the dense distribution records of sections *Ilex* and *Quercus* were filtered to ensure that the geographic records were even between main regions using the R packages raster [129] and dismo [129]. The filtering of section *Ilex* in west Europe retained only one occurrence record in each raster of 0.45 km × 0.45 km, but one record for each raster of 0.35 km × 0.35 km in SW China. The filtering resolution of section *Quercus* in Europe and North America retained one record in each raster of 0.04 km × 0.04 km. Records of each section were plotted on a map and the number of records were counted to ensure that the point densities were even between different regions.

Multicollinearity among 19 BIOCLIM variables was examined using a Pearson correlation matrix estimated in the R package psych [135]. The subsets of variables with high correlations (r > 0.8) were reduced to single variables. The restricted climatic variable for each section was calculated in MAXENT 3.4.1 [136] based on the reduced subsets of variables. Density curves of restricted climatic variables were plotted using the R package ggplot2 [137].

### Modeling the effect of geography and climate on chloroplast phylogeny

Nonmetric multidimensional scaling (NMDS) was used on all 19 BIOCLIM variables to characterize the climatic niche for the three oak sections (Additional file 1: Table S2). Ordination was conducted on a Gower distance matrix which could handle variables with different physical units and of mixed precision levels [138]. NMDS was conducted from K = 1 to K = 10, and stress was plotted to select the best-fit number of dimensions for ordination. For visualization, tree topologies were projected into the two-dimensional (K = 2) ordination space. Analyses were conducted using the R packages vegan [139] and phytools [127].

Mantel and multiple mantel tests were performed in the R package phytools [127] using the following regression models: genetic distances predicted by geographic distances (Δgenetic~Δgeo); genetic distances predicted by climatic distances (Δgenetic~Δclim); genetic variation predicted by geographic and climatic distances (Δgenetic~Δgeo +Δclim). GenAlEx 6.5 [124] was used to calculate the genetic distance and the geographic distance of each section. Gower distances of 19 BIOCLIM variables were calculated in R using vegan [139].

## Supplementary information

**Additional file 1.** Detailed information and data.

**Additional file 2. **Distribution of shared haplotypes of section *Ilex* (a) and section *Quercus* (b).

## Data Availability

DNA sequences are from references [50, 119]. Sampling distribution and climate data are uploaded as additional files.

## Abbreviations

SW: Southwest
