# Supplementary figures and images for: Ancient events and climate adaptive capacity shaped distinct chloroplast genetic structure in the oak lineages
Source: BMC Evol Biol. 2019 Nov 4;19:202. doi: 10.1186/s12862-019-1523-z (PMC6829957; doi:10.1186/s12862-019-1523-z)

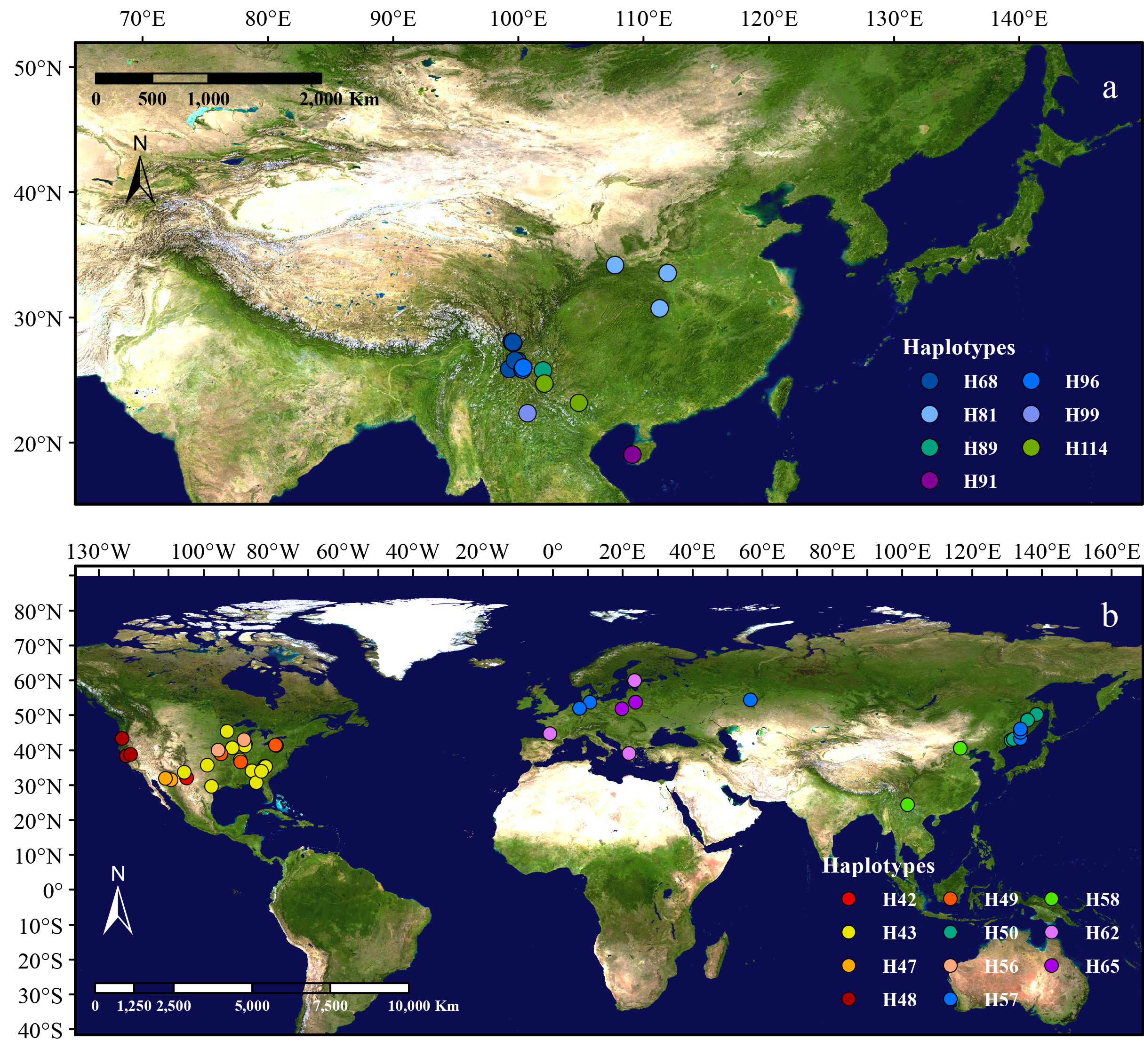

Supplement: Supplementary file 1 — Additional file 1. Detailed information and data. [file 12862_2019_1523_MOESM1_ESM.tif]

**
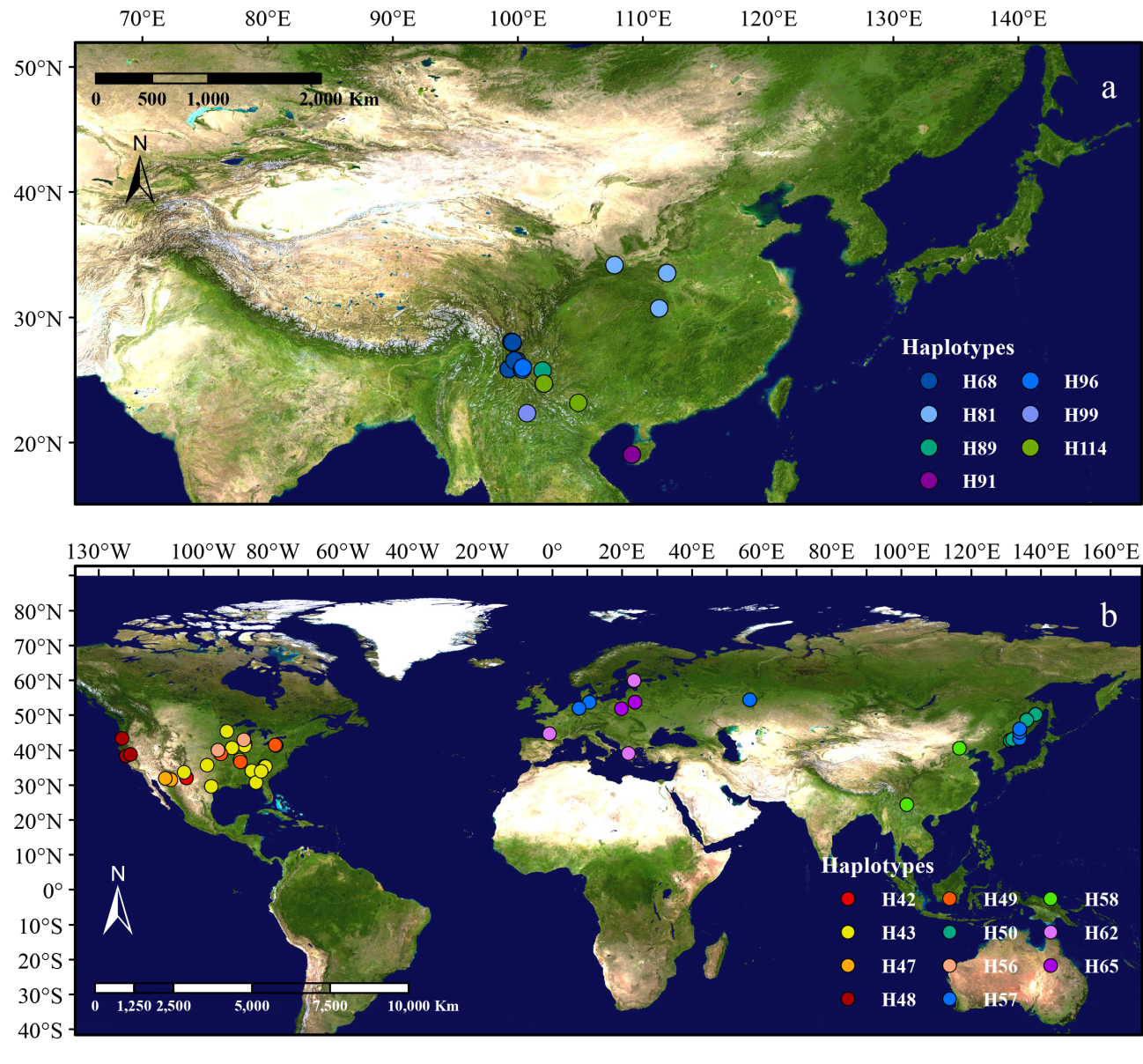
**

**Additional file 2** Distribution of shared haplotypes of section Ilex (a) and section Quercus (b).

Supplement: Supplementary file 2 — Additional file 2. Distribution of shared haplotypes of section Ilex (a) and section Quercus (b). [file 12862_2019_1523_MOESM2_ESM.docx]
